# The Role of Toll-Like Receptor 2 and 4 Innate Immunity Pathways in Intracortical Microelectrode-Induced Neuroinflammation

**DOI:** 10.3389/fbioe.2018.00113

**Published:** 2018-08-14

**Authors:** John K. Hermann, Shushen Lin, Arielle Soffer, Chun Wong, A. Ali, Jeremy Chang, Smrithi Sunil, Shruti Sudhakar, William H. Tomaszewski, Grace Protasiewicz, Stephen M. Selkirk, Robert H. Miller, Jeffrey R. Capadona

**Affiliations:** ^1^Department of Biomedical Engineering, Case Western Reserve University, Cleveland, OH, United States; ^2^Advanced Platform Technology Center, Louis Stokes Cleveland Veterans Affairs Medical Center, Cleveland, OH, United States; ^3^Department of Neurology, Case Western Reserve University, Cleveland, OH, United States; ^4^Spinal Cord Injury Division, Louis Stokes Cleveland Veterans Affairs Medical Center, Cleveland, OH, United States; ^5^Neurosciences, George Washington University, Washington, DC, United States

**Keywords:** BCI, intracortical microelectrode, neuroinflammation, innate immunity, Toll-like receptors

## Abstract

We have recently demonstrated that partial inhibition of the cluster of differentiation 14 (CD14) innate immunity co-receptor pathway improves the long-term performance of intracortical microelectrodes better than complete inhibition. We hypothesized that partial activation of the CD14 pathway was critical to a neuroprotective response to the injury associated with initial and sustained device implantation. Therefore, here we investigated the role of two innate immunity receptors that closely interact with CD14 in inflammatory activation. We implanted silicon planar non-recording neural probes into knockout mice lacking Toll-like receptor 2 (*Tlr2*^−/−^), knockout mice lacking Toll-like receptor 4 (*Tlr4*^−/−^), and wildtype (WT) control mice, and evaluated endpoint histology at 2 and 16 weeks after implantation. *Tlr4*^−/−^ mice exhibited significantly lower BBB permeability at acute and chronic time points, but also demonstrated significantly lower neuronal survival at the chronic time point. Inhibition of the Toll-like receptor 2 (TLR2) pathway had no significant effect compared to control animals. Additionally, when investigating the maturation of the neuroinflammatory response from 2 to 16 weeks, transgenic knockout mice exhibited similar histological trends to WT controls, except that knockout mice did not exhibit changes in microglia and macrophage activation over time. Together, our results indicate that complete genetic removal of Toll-like receptor 4 (TLR4) was detrimental to the integration of intracortical neural probes, while inhibition of TLR2 had no impact within the tests performed in this study. Therefore, approaches focusing on incomplete or acute inhibition of TLR4 may still improve intracortical microelectrode integration and long term recording performance.

## Introduction

Brain machine interfaces are a growing area of interest for basic research, rehabilitation, and commercial applications (Winkler, 2017; Wu and Rao, 2017; Savage, 2018). Intracortical microelectrodes remain a high-resolution tool for extracting information from the brain (Ajiboye et al., 2017), critical for current and future applications. Unfortunately, inconsistent recording performance remains a barrier to long-term utilization in any animal model, including humans (Gunasekera et al., 2015; Patel et al., 2016; Wellman et al., 2018).

The correlation between the neuroinflammatory response to intracortical microelectrodes and recording performance remains a commonly debated topic for over a decade (Szarowski et al., 2003; Biran et al., 2005; Jorfi et al., 2015). The consensus of the field is that in order to maintain viable recordings, the integrity of both the implanted electrodes and the neural tissue must remain intact. Several labs have shown that delamination of the insulation layers or corrosion of the electrode contacts are common in both accelerated aging and upon explanting of a variety of microelectrode types (Prasad et al., 2012, 2014; Barrese et al., 2013; Jorfi et al., 2015; Kozai et al., 2015; Takmakov et al., 2015). Additionally, the loss of neuronal cell bodies and dendrites within the distance required for single unit detection (Buzsáki, 2004) is well documented (Jorfi et al., 2015). While subtle difference exist across all microelectrode types, the typical response to intracortical microelectrodes can be generalized (Jorfi et al., 2015); upon implantation of the microelectrodes, tissue and cells are damaged resulting in both wound healing and scar formation. Most importantly, the robust response from microglia and macrophages leads to neuronal dieback, astrocytic encapsulation, and blood-brain barrier permeability, each of which have been implicated in biological failure mechanisms of single unit recordings from intracortical microelectrodes (Polikov et al., 2005; McConnell et al., 2009; Saxena et al., 2013; Jorfi et al., 2015).

As the failure modes of intracortical microelectrodes are further elucidated, one mechanism that has been suggested to play a key role in several failure modes is oxidative stress and/or Fenton chemical reactions (iron catalyzed peroxide formation) at the microelectrode-tissue interface (Prasad et al., 2012, 2014; Barrese et al., 2013; Potter et al., 2013, 2014; Potter-Baker et al., 2014a, 2015; Potter-Baker and Capadona, 2015; Ereifej et al., 2018). Pro-inflammatory cells (activated microglia, macrophages and astrocytes) remain reactive on and around the intracortical microelectrodes for the duration of implantation (McConnell et al., 2009; Ravikumar et al., 2014b; Nguyen et al., 2016). Furthermore, it is understood that these pro-inflammatory cells release cytokines (Polikov et al., 2005), free radicals, reactive oxygen species (ROS) and reactive nitrogen species (RNS) when activated (Streit et al., 1999; Abbott et al., 2006; Kettenmann et al., 2011).

Many attempts have been made to alter the design or materials properties of the intracortical microelectrodes to minimize the neuroinflammatory response (for review see Jorfi et al., 2015). We have utilized many antioxidative strategies to specifically attenuate oxidative damage, resulting in higher densities of neuronal nuclei and more viable neurons at the intracortical microelectrode / tissue interface (Potter et al., 2013, 2014; Potter-Baker et al., 2014a; Jorfi et al., 2015; Nguyen et al., 2016). In parallel, we have also attempted to understand the subcellular mechanisms at play in the initiation of reactive oxygen species generation, in response to the implantation and chronic indwelling of intracortical microelectrodes (Ereifej et al., 2018).

In that respect, we have identified the innate immunity receptor CD14 as a molecule of interest in the chronic neuroinflammatory response to implanted intracortical microelectrodes (Bedell et al., 2018; Hermann et al., 2018). CD14 is a molecule associated with the recognition of pathogen associated molecular patterns (PAMPs) and damage associated molecular patterns (DAMPs) to promote inflammation, including the release of numerous cytokines, chemokines, and reactive oxygen species (Reed-Geaghan et al., 2009; Janova et al., 2015). Hermann et al. first observed acute but not chronic improvements in intracortical microelectrode recording performance in knockout mice lacking CD14, and chronic improvements in recording performance in mice receiving a small-molecule inhibitor to the CD14 pathway (Hermann et al., 2018). More recently, using a bone marrow chimera model, Bedell et al. demonstrated that inhibiting CD14 from only the blood-derived macrophages, and not resident brain derived glial cells improves recording quality over the 16 week long study (Bedell et al., 2018). Together, these two studies indicated that partial inhibition of CD14 pathways resulted in a greater improvement to microelectrode performance than complete inhibition. Therefore, we are interested in developing a better understanding of the mechanism, to optimize the natural wound healing response, yet still inhibit the over-excitation of the pathway that can lead to decreased microelectrode performance.

In the current study, we will focus on the complementary receptors associated with CD14 activation, Toll-like receptors 2 and 4 (TLR2 and TLR4). TLR4 is an innate immunity receptor closely associated with CD14 that is involved in the recognition of PAMPs and DAMPs to promote inflammation (Asea et al., 2002; Reed-Geaghan et al., 2009; Trotta et al., 2014). TLR2 is another innate immunity receptor closely associated with CD14 that is involved in the recognition of PAMPs and DAMPs to promote inflammation (Asea et al., 2002; Reed-Geaghan et al., 2009; Piccinini and Midwood, 2010; Kong and Le, 2011). Both TLR2 and TLR4 have been associated with neurodegenerative disorders (Landreth and Reed-Geaghan, 2009; Arroyo et al., 2011; Casula et al., 2011; Kong and Le, 2011; Trotta et al., 2014). Of note, the small molecule inhibitor to CD14 (IAXO-101, Innaxon) is also listed as a TLR4 inhibitor. Thus, we hypothesize that TLR2 and TLR4 differentially play a role in the neuroinflammatory response to implanted intracortical microelectrodes. To test this hypothesis, we implanted silicon planar non-recording neural probes in the shape of Michigan-style intracortical microelectrode arrays into knockout mice lacking TLR2 (*Tlr2*^−/−^), knockout mice lacking TLR4 (*Tlr4*^−/−^), and WT control mice, and evaluated endpoint histology at 2 and 16 weeks after implantation. Gaining a more detailed understanding of innate immunity receptors associated with the CD14 pathway should further our understanding of CD14 mediated neuroinflammation to intracortical microelectrodes.

## Materials and methods

### Animal model

*Tlr2*^−/−^ mice (B6.129-Tlr2^tm1Kir^/J, stock no. 004650), *Tlr4*^−/−^ mice (B6.B10ScN-Tlr4^lps−del^/JthJ, stock no. 007227), and WT mice (C57BL/6J, stock no. 000664) were acquired from the Jackson laboratory and bred in-house. Both male and female mice were used as to not bias the results based on sex. Mice were handled according to the approved Case Western Reserve University IACUC protocol and the NIH Guide for Care and Use of Laboratory Animals.

### Genotyping

Strains of mice were verified prior to surgery by extracting DNA from tail snips, running PCR, and running gel electrophoresis. Genotyping protocols were performed as suggested by the mouse vendor (Jackson Laboratories), following similar protocols described in previous studies within the lab (Bedell et al., 2018).

### Probe implantation surgery

Mice were implanted with neural probes (described in detail below) using methods adapted from Ravikumar et al. (2014a,b) and Potter-Baker et al. (2014b). Mice were aged to between 5 and 9 weeks; and weighed between 14 and 29 grams at the time of surgery. Each mouse was induced with 3% isoflurane in an induction chamber. While under anesthesia, mice were mounted to the ear bars of the stereotaxic frame, and anesthesia was lowered to 1% isoflurane for maintenance. Mice were kept on a heating pad while under anesthesia to maintain body temperature. Ophthalmic ointment was applied to the eyes of the mouse to prevent drying, followed by shaving of the scalp with a hand-held beard trimmer. The scalp of the mouse was sterilized with three applications of betadine alternated with 70% isopropanol. Marcaine (0.02 ml, 0.25%) was injected below the scalp, at the surgical site, as a local anesthetic. Either buprenorphine (0.1 mg/kg) or meloxicam (0.07 ml, 1.5 mg/mL) were administered subcutaneously as an analgesic. Choice of analgesic changed during the study due to availability from the vendor at the time. Substitutions were chosen following consultation with staff veterinarian. Additionally, cefazolin (0.2 mL, 2 mg/mL) was injected subcutaneously as an antibiotic, to prevent post-operative infection. The proper surgical plane of anesthesia was verified using a toe pinch throughout the surgery.

Mouse skulls were exposed by a midline incision on the scalp and retraction of the skin using tissue spreaders. Craniotomies were carried out using a 3 mm biopsy punch (PSS select) lateral to midline, and between lambda and bregma, to minimize heat associated with drilling (Shoffstall et al., 2017). One alcohol cleaned, ethylene oxide-sterilized silicon probe in the shape of a Michigan-style microelectrode array (2 mm long × 123 μm wide (tapered) × 15 μm thick, 1 mm x 1 mm bond tab) was inserted 2 mm into the craniotomy via forceps to avoid vasculature. Prior to sterilization, electrodes were cleaned in 70% ethanol and de-ionized water. Craniotomies were sealed with a biocompatible silicone elastomer (Kwik-sil) and closed with a UV-cured liquid dentin (Fusio/Flow-it ALC, Pentron dental). Protruding bond tabs were encased in the liquid dentin to anchor the implant. Incisions were sutured closed with 5-0 monofilament polypropylene suture (Butler Schein). Antibiotic ointment was applied to the incision to prevent infection.

Mice were administered cefazolin (0.2 mL, 2 mg/mL) subcutaneously twice on the first day after surgery. Mice were administered meloxicam (0.07 ml, 1.5 mg/mL) once or buprenorphine (0.1 mg/kg) twice on the first day after surgery and as needed thereafter. Mice were monitored daily for 5 days after the operation for signs of pain and distress and then weekly thereafter.

### Tissue processing

Mice were transcardially perfused 2 or 16 weeks after probe implantation using protocols adapted from Ravikumar et al. (2014a). Prior to transcardial perfusion, mice were anesthetized with 0.3 ml of a 10 mg ml^−1^ ketamine, 1 mg ml^−1^ xylazine solution, administered subcutaneously. Additional ketamine-xylazine solution was administered as needed. Following perfusions, the mouse heads were removed and post-fixed in 4% paraformaldehyde dissolved in 1xPBS overnight. Liquid dentin skull caps were carefully removed up from the skulls to remove implanted electrodes and minimize damage to the tissue. Brains were gently extracted from skulls and transferred to a series of sucrose solutions with concentrations of 10%, 20%, and two rounds of 30% in 1xPBS for cryoprotection. Upon equilibration (typically overnight), brains were advanced to the next solution in the series and stored at 4°C. Next, brains were frozen in blocks of Optical Cutting Temperature gel over dry ice and moved to a −80°C freezer. Finally, brains were cryostat sectioned into 16 μm horizontal slices and directly mounted to slides at a ~ −20 to −25°C, and stored at −80°C until removed for immunohistochemical staining.

### Endpoint histology

Two cohorts of animals were implanted with neural probes to assess both the acute and chronic neuroinflammatory response to implanted intracortical microelectrodes. For the purposes of this study, four histological markers were investigated. First, since neurons are the source of electrical signals recorded by intracortical microelectrodes, sections of cortical tissue were stained with an antibody directed against NeuN, a nuclear protein specific to neurons (Mullen et al., 1992). Neuronal dieback around implanted intracortical microelectrode arrays is commonly attributed to the release of soluble factors by inflammatory activated microglia and macrophages (Polikov et al., 2005). Therefore, to understand how the absences of TLR2 and TLR4 affect inflammatory activation of microglia and macrophages in response to implanted neural probes, sections of cortical tissue were stained with an antibody directed against CD68 (macrosialin), a sialoglycoprotein found in activated microglia and macrophages (Rabinowitz and Gordon, 1991). Another byproduct of chronic inflammatory mechanisms potentially detrimental to intracortical microelectrode activation is astrocytic encapsulation. Astrocytes may become hypertrophic in response to implanted microelectrode arrays and encase the array in a sheath that impedes electrical signals (Burns et al., 1974). Therefore, to understand how the absences of TLR2 and TLR4 affect astrocytic encapsulation in response to implanted neural probes, sections of cortical tissue were stained with an antibody directed against glial fibrillary acidic protein (GFAP), an astrocytic intermediate filament that is upregulated during inflammatory activation (Eddleston and Mucke, 1993). Finally, another component of chronic inflammatory mechanisms correlated to poor recording performance is blood-brain barrier permeability (Saxena et al., 2013). To understand how the absences of TLR2 and TLR4 affect blood-brain barrier permeability in response to implanted neural probes, sections of cortical tissue were stained with an antibody directed against IgG, a blood protein not normally found in healthy brain tissue.

#### Immunohistochemical staining

Staining protocols were adapted from Ravikumar et al. (2014a). Microscope slides were removed from the freezer and equilibrated to room temperature in a humidity chamber. Brain slices were blocked in buffers composed of 4% goat or chicken serum, 0.3% Triton-X 100, and 0.1% sodium azide dissolved in 1x PBS for 1 h at room temperature. Primary and secondary stain selections and dilutions are summarized in Table 1. Brain slices were incubated in primary antibody solutions dissolved in blocking buffers containing serum matching the species of secondary antibody. Brain slices were incubated in secondary antibody solutions for 2 h at room temperature. Tissue auto-fluorescence was reduced via the application of a copper sulfate solution, as described by Potter et al. (2012b). Microscope slides of brain slices were coverslipped using fluromount-G mounting medium.

**Table 1:** Summary of immunohistochemical reagents used in histology.

**Target**	**Antigen**	**1^°^Antibody**	**Concentration**	**2^°^Antibody/Detection Kit**	**Concentration**
Neurons (DAB)	NeuN	Millipore MAB377	1:250	Life Technologies 879163	N/A
Neurons (Fluorescent)	NeuN	Millipore MAB377	1:250	Thermo Fisher Scientific A21121	1:1,000
Astrocytes	GFAP	Neuromics RA22101	1:500	Thermo Fisher Scientific A11012	1:1,000
Activated Microglia/Macrophages	CD68	Abcam ab53444	1:500	Thermo Fisher Scientific A21470	1:1,000
Extravasated Blood Proteins	IgG	AbD Serotec STAR26B	1:500	Thermo Fisher Scientific A21442	1:1,000

#### DAB staining for neuronal nuclei

A majority of the tissue slices stained for neuronal nuclei using NeuN were followed with a DAB chromogen to make the cells visible under brightfield light. Protocols to stain neuronal nuclei were adapted from Ravikumar et al. (2014a). Brain slices were blocked in goat blocking buffer for 1 h at room temperature. Slices were then incubated in blocking buffer solutions containing NeuN antibodies (Millipore MAB377) diluted 1:250 for 1 h at room temperature. Horseradish peroxidase polymer and DAB chromogen were added to the brain slices according to the manufacturer protocols (Life Technologies Super PicTure Polymer Detection Kit, Ref 879163). Hematoxylin was applied to the brain slices as a counter stain for all cell nuclei. Slides were coverslipped with Histomount mounting medium.

Additional NeuN sections were added using a superior protocol involving a fluorescent secondary antibody. These sections were stained following the protocols established in section Endpoint Histology. Since NeuN was quantified by hand, regardless of the protocol, no difference in results were achieved with the two protocols, just ease of quantification for the counter.

### Imaging

Fluorescent images were captured using a Zeiss AxioObserver Z1 inverted fluorescent microscope with a 10x objective and an AxioCam MRm monochrome camera. As described in Hermann et al. 4 × 4 mosaic images centered on the probe hole were assembled using AxioVision and Zen software (Hermann et al., 2018).

Color images were captured for NeuN stains utilizing a DAB chromogen, as described by Ravikumar et al. (2014a). The same AxioObserver Z1 inverted microscope was used with a 10x objective and an Erc5 color camera. As described by Ravikumar et al. 4 × 4 mosaic images were assembled using Axiovision software (Ravikumar et al., 2014a).

**Figure 1:**
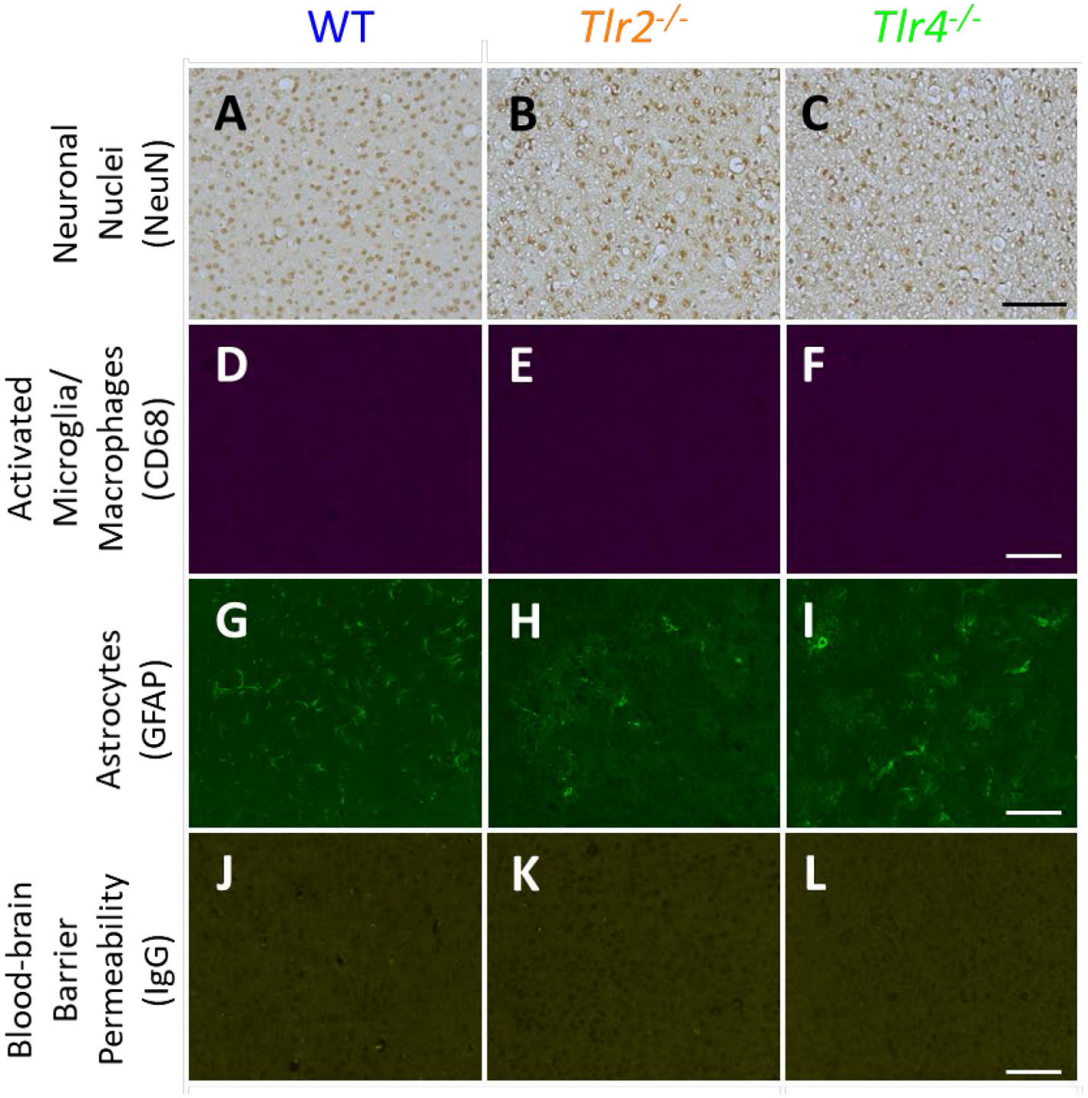
Immunohistochemical staining in sham animals. WT **(A,D,G,J)**, *Tlr2*^−/−^**(B,E,H,K)**, and *Tlr4*^−/−^ **(C,F,I,L)** mice without implanted probes were stained for neuronal nuclei (NeuN, **A-C**), activated microglia and macrophages (CD68, **D–F**), astrocytes (GFAP, **G–I**), and blood-brain barrier permeability (IgG, **J–L**). Scale bars are provided for each row of images, scale = 100 μm.

Representative images of sham animals not implanted with neural probes are displayed in Figure 1. Sham animals were age matched to the 16 week group and underwent the same perfusion, tissue processing, and staining protocols as described above. This figure demonstrates constitutive expression of NeuN, CD68, GFAP, and IgG in *Tlr2*^−/−^, *Tlr4*^−/−^, and WT mice. No differences were seen in control, non-implanted shams.

The brightness and color balance of representative images were adjusted to enhance visibility.

### Neuronal nuclei counting

Neuronal nuclei counts were obtained using our freely available custom Matlab scripts Second and AfterNeuN as described in Hermann et al. (2018). A user traced the outline of the probe hole and defined artifacts (tissue edge, etc) using Second and subsequently defined NeuN positive cell positions using AfterNeuN. Users counted out to a background defined as 400 μm from the probe hole plus a 50 μm buffer zone. Neuronal density was quantified as cell count per area, in concentric 50 μm bins extending from the probe hole. Percent of background density was quantified as bin neuronal density divided by the neuronal density of the 300–400 μm region times 100. Average percent of background was assessed for each animal using 3–7 slices. Mean and standard error were defined for each group based on animal averages from 4 to 7 mice per group. Statistics are calculated using animal averages.

### Immunohistochemical marker quantification

Immunohistochemical markers were quantified using the Matlab script Second, as described in Hermann et al. (2018). A user traced the outline of the probe hole and defined artifacts using Second. Second calculated fluorescence intensity in 5 μm concentric bins out to a distance of 650 μm from the probe. Background intensity was defined as the fluorescence intensity in the 600–650 μm region. Fluorescence intensity was normalized to a value of 0 at background for CD68 and IgG as no CD68 or IgG expression is seen in the non-implanted sham (Figure 1), and normalized to a value of 1 at background for GFAP, as GFAP is expressed in native tissue (Figure 1). Area under the curve was calculated for 50 μm bins extending away from the probe hole; 5 μm normalized intensity bins and 50 μm area under the curve bins were averaged by animal across 3–6 slices. Mean and standard error for each group were based on animal averages from 4 to 8 mice per group. Statistics were calculated using animal averages of 50 μm area under the curve bins.

### Statistics

All statistical comparisons were carried out using Minitab software. For each time point neuronal nuclei percent background density and normalized fluorescence intensity area under the curve animal averages are compared between knockout mice and WT controls. Animal average values for a given stain were entered into a general linear model with animal average values from other strains at the same time point. Comparisons are made between *Tlr2*^−/−^ and WT mice and between *Tlr4*^−/−^ and WT mice using a Bonferroni test. Significance was defined as a p value less than 0.05.

Additionally, statistical comparisons were made between mice of the same strain at different time points. Neuronal nuclei percent background density and normalized fluorescence intensity area under the curve animal averages are compared between 2 and 16 week time points. Animal average values from a given strain and time point are entered into a general linear model with animal average values from the same strain at the opposite time point. Comparisons are made between 2 and 16 week mice for a given strain using a Bonferroni test. Significance was defined as a p value less than 0.05.

## Results

### Acute (2-week) neuroinflammatory response to implanted intracortical probes in *Tlr2^−/−^, Tlr4^−/−^*, and WT mice

**Figure 2:**
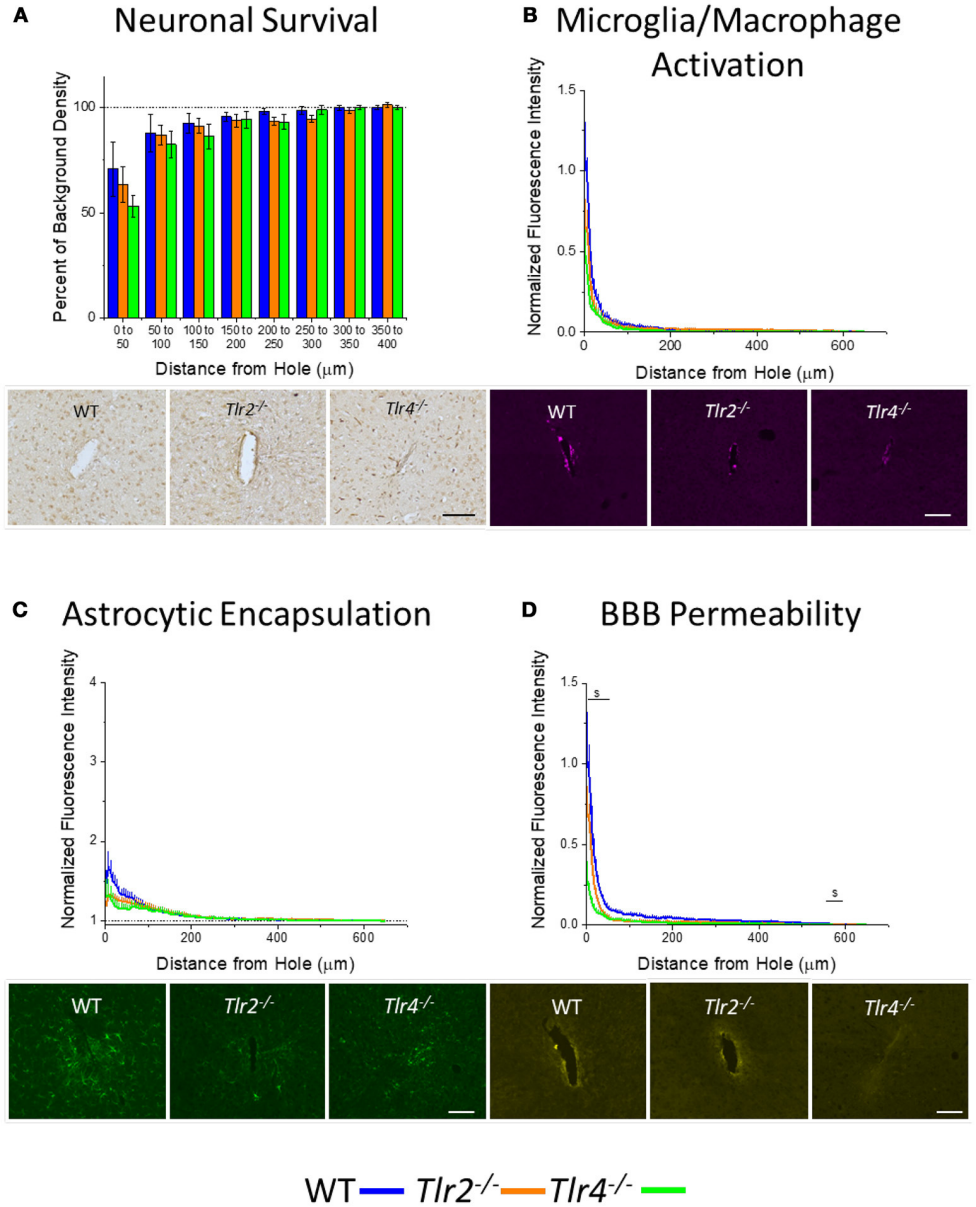
Acute immunohistochemical evaluation of *Tlr2*^−/−^, *Tlr4*^−/−^, and wildtype mice two weeks after probe implantation. **(A)** Neuronal survival in cortical tissue around implanted probes presented as percent of background density with respect to distance from probe hole (μm). No significant differences were observed between either of the knockout conditions and wildtype mice. *Tlr2*^−/−^: *N* = 7; *Tlr4*^−/−^: *N* = 5; WT: *N* = 5. **(B)** Microglia and macrophage activation in cortical tissue around implanted probes presented as normalized fluorescence intensity with respect to distance from probe hole (μm). Normalized fluorescence intensity indicates expression of CD68. No significant differences were observed between either of the knockout conditions and wildtype mice. *Tlr2*^−/−^: *N* = 8; *Tlr4*^−/−^: *N* = 4; WT: *N* = 6. **(C)** Astrocyte encapsulation in cortical tissue around implanted probes presented as normalized fluorescence intensity with respect to distance from probe hole (μm). Normalized fluorescence intensity indicates expression of GFAP. No significant differences were observed between either of the knockout conditions and wildtype mice. *Tlr2*^−/−^: *N* = 8; *Tlr4*^−/−^: *N* = 4; WT: *N* = 6. **(D)** Blood-brain barrier permeability presented as normalized fluorescence intensity with respect to distance from probe hole (μm). Normalized fluorescence intensity indicates expression of IgG. *Tlr4*^−/−^ mice exhibited significantly less IgG expression within distances of 0–50 and 550–600 μm from the probe hole, indicating reduced blood-brain barrier permeability, $ *p* < 0.05. *Tlr2*^−/−^: *N* = 5; *Tlr4*^−/−^: *N* = 4; WT: *N* = 6. Orange = *Tlr2*^−/−^, Green = *Tlr4*^−/−^, and Blue = WT. Scale bars are provided for each set of images, scale = 100 μm.

Plots of percentage of background neuronal density (Neuronal survival) or normalized fluorescence intensity (microglia/macrophage activation, astrocytic encapsulation, blood-brain barrier permeability) with respect to distance from probe hole for *Tlr2*^−/−^, *Tlr4*^−/−^, and WT are shown in Figure 2. All three strains of mice exhibited trends of increasing neuronal density, decreasing microglia/macrophage activation, decreasing astrocytic encapsulation, and decreasing blood-brain barrier permeability with increasing distance from the probe hole at the acute 2-week time point. Examination of neuronal survival via the NeuN stain in *Tlr2*^−/−^, *Tlr4*^−/−^, and WT mice (Figure 2A) revealed no statistical differences between groups at the counted distance intervals (*Tlr2*^−/−^: *N* = 7; *Tlr4*^−/−^: *N* = 5; WT: *N* = 5). Similarly, examination of the accumulation of inflammatory activated microglia and macrophages via CD68 revealed no significant differences between either of the knockout conditions and WT mice (*Tlr2*^−/−^: *N* = 8; *Tlr4*^−/−^: *N* = 4; WT: *N* = 6). Additionally, examination of the chronic glial scar as a function of GFAP expression also indicated no significant differences were observed between either of the knockout conditions and WT mice (Figure 2C). Unlike glial cell density, blood-brain barrier permeability (as a function of IgG expression) indicated significant differences between experimental and control group at the acute 2-week time point (Figure 2D). Specifically, *Tlr4*^−/−^ mice exhibit significantly less IgG expression compared to WT mice at the distance intervals 0–50 and 550–600 μm from the probe hole, indicating reduced blood-brain barrier permeability, $*p* < 0.05 (*Tlr2*^−/−^: *N* = 5; *Tlr4*^−/−^: *N* = 4; WT: *N* = 6). *Tlr2*^−/−^ mice did not exhibit any differences in IgG expression from WT controls.

### Chronic (16-week) neuroinflammatory response to implanted intracortical probes in *Tlr2^−/−^, Tlr4^−/−^*, and WT mice

**Figure 3:**
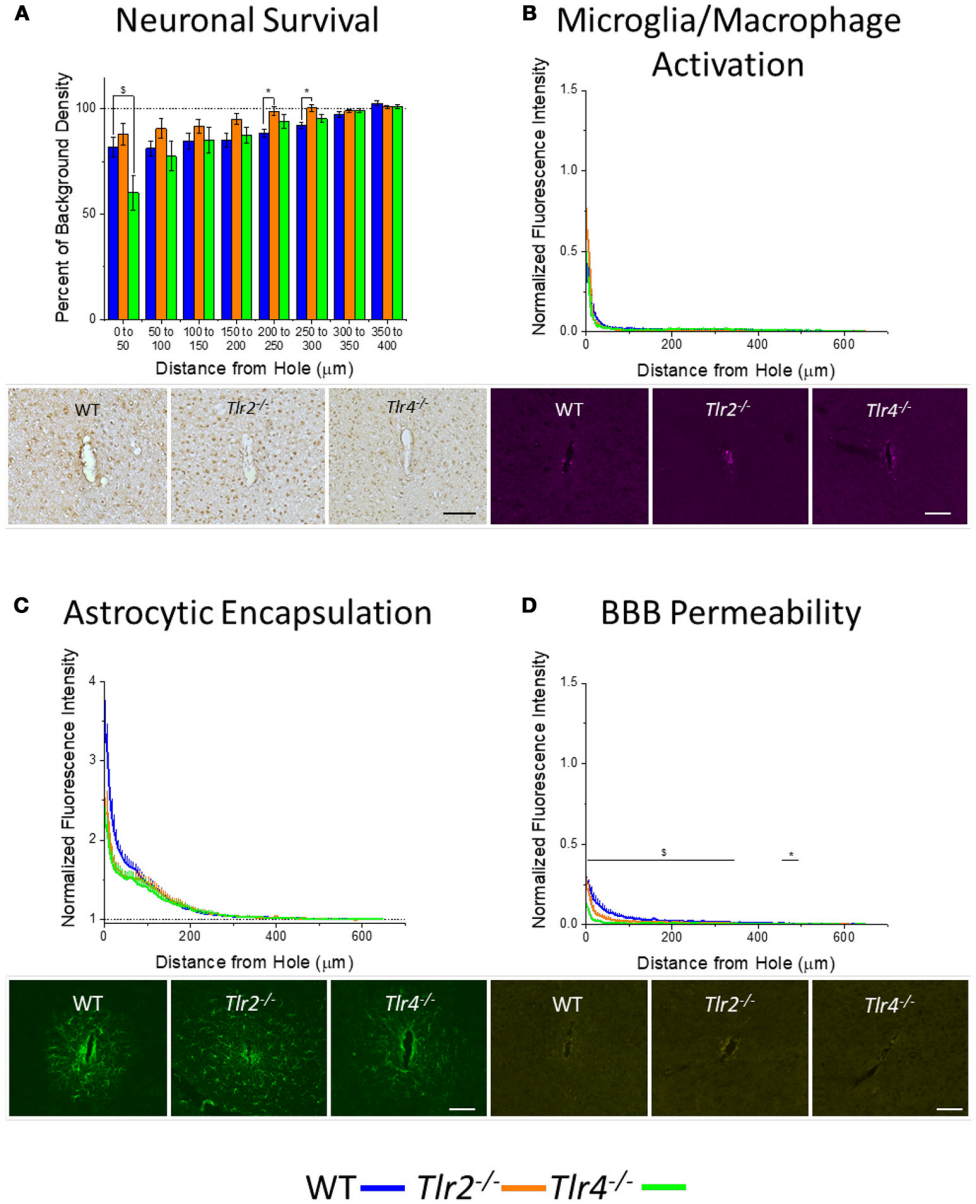
Chronic immunohistochemical evaluation of *Tlr2*^−/−^*, Tlr4*^−/−^, and *wildtype* mice 16 weeks after probe implantation. **(A)** Neuronal survival in cortical tissue around implanted probes presented as percent of background density with respect to distance from probe hole (μm). Neuronal survival in *Tlr4*^−/−^ mice was significantly lower than neuronal survival in wildtype mice in the distance interval 0–50 μm from the probe hole. Neuronal survival in *Tlr2*^−/−^ mice was significantly higher than neuronal survival in wildtype mice in the distance intervals 200–250 and 250–300 μm from the probe hole. *Tlr2*^−/−^: *N* = 5; *Tlr4*^−/−^: *N* = 5; WT: *N* = 7. **(B)** Microglia and macrophage activation in cortical tissue around implanted probes presented as normalized fluorescence intensity with respect to distance from probe hole (μm). Normalized fluorescence intensity indicates expression of CD68. No significant differences were observed between either of the knockout conditions and wildtype mice. *Tlr2*^−/−^: *N* = 5; *Tlr4*^−/−^: *N* = 5; WT: *N* = 7. **(C)** Astrocyte encapsulation in cortical tissue around implanted probes presented as normalized fluorescence intensity with respect to distance from probe hole (μm). Normalized fluorescence intensity indicates expression of GFAP. No significant differences were observed between either of the knockout conditions and wildtype mice. *Tlr2*^−/−^: *N* = 5; *Tlr4*^−/−^: *N* = 5; WT: *N* = 7. **(D)** Blood-brain barrier permeability presented as normalized fluorescence intensity with respect to distance from probe hole (μm). Normalized fluorescence intensity indicates expression of IgG. *Tlr4*^−/−^ mice exhibited significantly less IgG expression within each distance interval examined from of 0-350 μm from the probe hole, indicating reduced blood-brain barrier permeability, $ *p* < 0.05. Additionally, *Tlr2*^−/−^ mice exhibited significantly less IgG expression within a distance of 450–500 μm from the probe hole, indicating reduced blood-brain barrier permeability, *p < 0.05. *Tlr2*^−/−^: *N* = 5; *Tlr4*^−/−^: *N* = 5; WT: *N* = 7. *Tlr2*^−/−^: *N* = 5; *Tlr4*^−/−^: *N* = 4; WT: *N* = 6. Orange = *Tlr2*^−/−^, Green = *Tlr4*^−/−^, and Blue = WT. Scale bars are provided for each set of images, scale = 100 μm.

An additional cohort of animals was implanted with identical non-functional probes for 16 weeks, to assess the chronic neuroinflammatory response (Potter et al., 2012a). Examination of neuronal density via the NeuN stain in *Tlr2*^−/−^*, Tlr4*^−/−^, and WT mice revealed a trend of increasing neuronal survival with distance from the probe at the 16 week time point (Figure 3A). Neuronal survival in *Tlr4*^−/−^ mice was significantly lower than neuronal survival in WT mice in the distance interval 0–50 μm from the probe hole. Neuronal survival in *Tlr2*^−/−^ mice was significantly higher than neuronal survival in WT mice in the distance intervals 200–250 and 250–300 μm from the probe hole (*Tlr2*^−/−^: *N* = 5; *Tlr4*^−/−^: *N* = 5; WT: *N* = 7). Examination of the accumulation of inflammatory activated microglia and macrophages via CD68 expression indicated that *Tlr2*^−/−^*, Tlr4*^−/−^, and WT mice all exhibit a trend of decreasing CD68 expression with distance from the probe hole at the chronic 16 week time point (Figure 3B). However, no significant differences were observed between either of the knockout conditions and WT mice (*Tlr2*^−/−^: *N* = 5; *Tlr4*^−/−^: *N* = 5; WT: *N* = 7). Similarly, examination of the chronic glial scar as a function of GFAP expression also indicated no significant differences between either of the knockout conditions and WT mice (*Tlr2*^−/−^: *N* = 8; *Tlr4*^−/−^: *N* = 4; WT: *N* = 6) (Figure 3C). Unlike glial cell density, blood-brain barrier permeability as a function of IgG expression indicated significant differences between experimental and control group at 16 weeks post-implantation (Figure 3D). Specifically, *Tlr4*^−/−^ mice exhibit significantly less IgG expression compared to WT mice at each of the interval examined from 0 to 350 μm from the probe hole, indicating reduced blood-brain barrier permeability, $*p* < 0.05. Additionally, *Tlr2*^−/−^ mice exhibit significantly less IgG expression within a distance of 450–500 μm from the probe hole, indicating reduced blood-brain barrier permeability, ^*^p < 0.05 (*Tlr2*^−/−^: *N* = 5; *Tlr4*^−/−^: *N* = 4; WT: *N* = 6).

### The progression of neuroinflammation and neurodegeneration following intracortical microelectrode implantation

**Figure 4:**
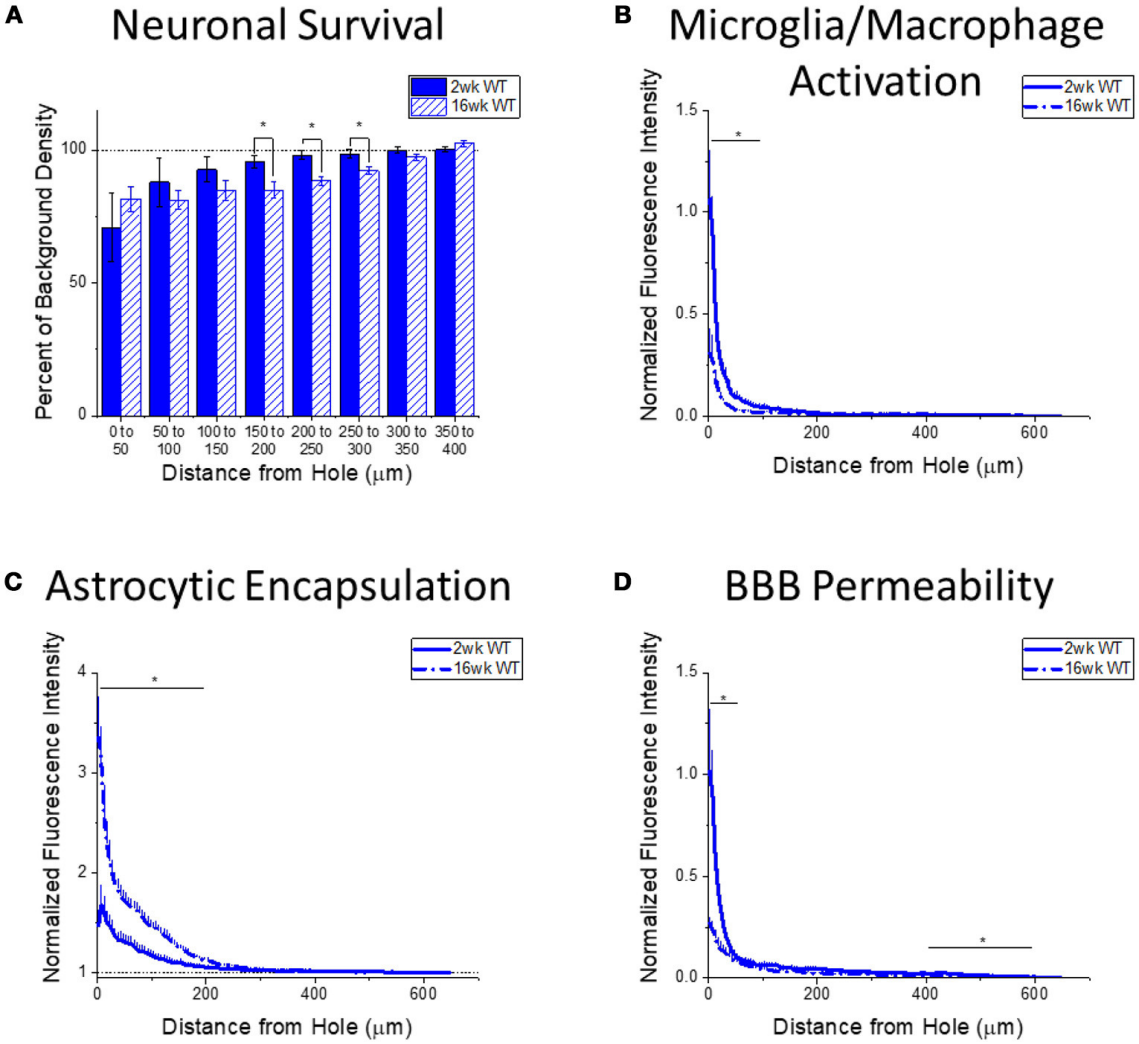
Changes in immunohistochemical markers in wildtype mice over time. **(A–D)** Show immunohistochemical marker expression in WT mice at 2 and 16 weeks after probe implantation. **(A)** Neuronal survival displayed as percent of background neuronal density with respect to distance from the probe hole (μm). Wildtype mice exhibit significantly higher neuronal survival at 2 weeks after implantation in distance intervals 150–200, 200–250, and 250–300 μm from the probe hole, *p < 0.05. 2 wk WT: *N* = 5; 16 wk WT: *N* = 7. **(B)** Microglia and macrophage activation (CD68) displayed as normalized fluorescence intensity with respect to distance from the probe hole (μm). Wildtype mice exhibited significantly higher microglia and macrophage activation at 2 weeks after probe implantation at distance intervals 0–50 and 50–100 μm from the probe hole, *p < 0.05. 2 wk WT: *N* = 6; 16 wk WT: *N* = 7. **(C)** Astrocytic encapsulation (GFAP) displayed as normalized fluorescence intensity with respect to distance from the probe hole (μm). Wildtype mice exhibit significantly higher astrocytic encapsulation at 16 weeks after probe implantation at distance intervals 0–50, 50–100, 100–150, and 150–200 μm from the probe hole, *p < 0.05. 2 wk WT: *N* = 6; 16 wk WT: *N* = 7. **(D)** Blood-brain barrier permeability (IgG) as normalized fluorescence intensity with respect to distance from the probe hole (μm). Wildtype mice exhibit significantly higher blood-brain barrier permeability at 2 weeks after probe implantation at distance intervals 0–50, 400–450, 450–500, 500–550, and 550–600 μm from the probe hole, *p < 0.05. 2 wk WT: *N* = 6; 16 wk WT: *N* = 7.

**Figure 5:**
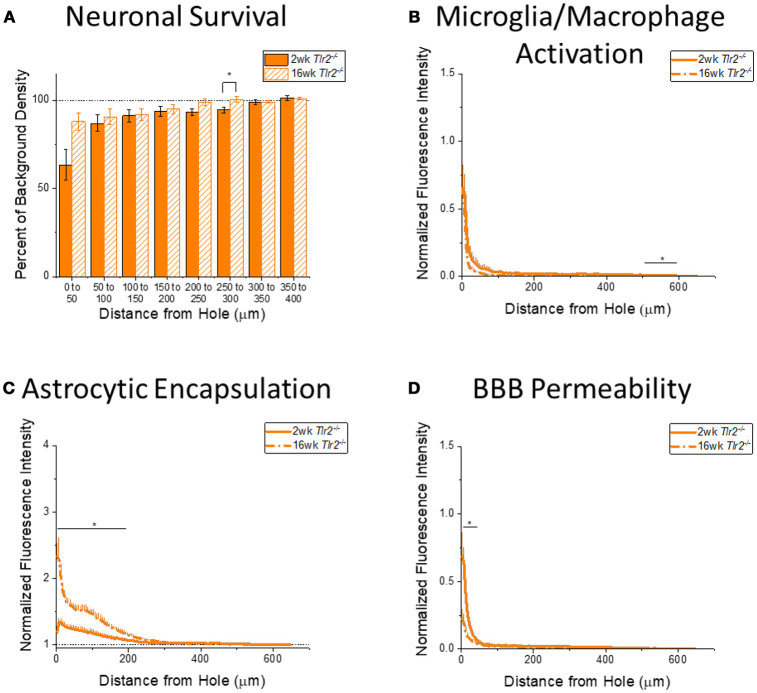
Changes in immunohistochemical markers in *Tlr2*^−/−^ mice over time. **(A–D)** Show immunohistochemical marker expression in *Tlr2*^−/−^ mice at 2 and 16 weeks after probe implantation. **(A)** Neuronal survival displayed as percent of background neuronal density with respect to distance from the probe hole (μm). *Tlr2*^−/−^ mice exhibit significantly higher neuronal survival at 16 weeks after probe implantation in the distance interval 250–300 μm from the probe hole. 2 wk *Tlr2*^−/−^: *N* = 7; 16 wk *Tlr2*^−/−^: *N* = 5. **(B)** Microglia and macrophage activation (CD68) displayed as normalized fluorescence intensity with respect to distance from the probe hole (μm). *Tlr2*^−/−^ mice exhibit significantly higher microglia and macrophage activation at 2 weeks after probe implantation at the distance interval 550–600 μm from the probe hole *p < 0.05. 2 wk *Tlr2*^−/−^: *N* = 8; 16 wk *Tlr2*^−/−^: *N* = 5. **(C)** Astrocytic encapsulation (GFAP) displayed as normalized fluorescence intensity with respect to distance from the probe hole (μm). *Tlr2*^−/−^ mice exhibit significantly higher astrocytic encapsulation at 16 weeks after probe implantation at distance intervals 0–50, 50–100, 100–150, and 150–200 μm from the probe hole, *p < 0.05. 2 wk *Tlr2*^−/−^: *N* = 8; 16 wk *Tlr2*^−/−^: *N* = 5. **(D)** Blood-brain barrier permeability (IgG) as normalized fluorescence intensity with respect to distance from the probe hole (μm). *Tlr2*^−/−^ mice exhibit significantly higher blood-brain barrier permeability at 2 weeks after probe implantation at the distance interval 0–50 μm from the probe hole, *p < 0.05. 2 wk *Tlr2*^−/−^: *N* = 5; 16 wk *Tlr2*^−/−^: *N* = 5.

**Figure 6:**
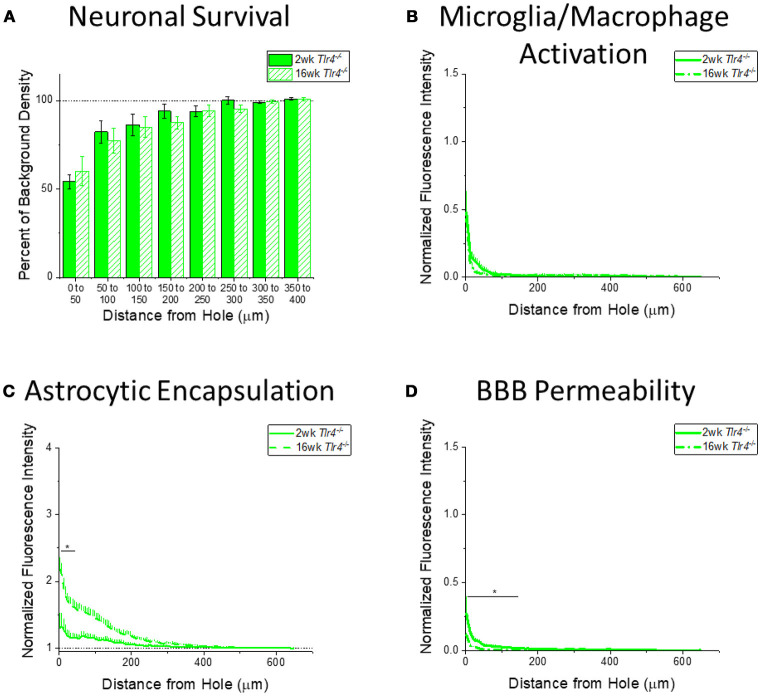
Changes in immunohistochemical markers in *Tlr4*^−/−^ mice over time. **(A–D)** Show immunohistochemical marker expression in *Tlr4*^−/−^ mice at 2 and 16 weeks after probe implantation. **(A)** Neuronal survival displayed as percent of background neuronal density with respect to distance from the probe hole (μm). *Tlr4*^−/−^ mice exhibit no significant difference in neuronal survival between two and 16 weeks after probe implantation. 2 wk *Tlr4*^−/−^: *N* = 5; 16 wk *Tlr4*^−/−^: *N* = 5. **(B)** Microglia and macrophage activation (CD68) displayed as normalized fluorescence intensity with respect to distance from the probe hole (μm). *Tlr4*^−/−^ mice exhibit no significant differences in microglia and macrophage activation between time points. 2 wk *Tlr4*^−/−^: *N* = 4; 16 wk *Tlr4*^−/−^: *N* = 5. **(C)** Astrocytic encapsulation (GFAP) displayed as normalized fluorescence intensity with respect to distance from the probe hole (μm). *Tlr4*^−/−^ mice exhibit significantly higher astrocytic encapsulation at 16 weeks after probe implantation the distance intervals 0–50 μm from the probe hole, *p < 0.05. 2 wk *Tlr4*^−/−^: *N* = 4; 16 wk *Tlr4*^−/−^: *N* = 5. **(D)** Blood-brain barrier permeability (IgG) as normalized fluorescence intensity with respect to distance from the probe hole (μm). *Tlr4*^−/−^ mice exhibit significantly higher blood-brain barrier permeability at 2 weeks after probe implantation at the distance intervals 0–50, 50–100, and100–150 μm from the probe hole, *p < 0.05. 2 wk *Tlr4*^−/−^: *N* = 4; 16 wk *Tlr4*^−/−^: *N* = 5.

It is also important to understand how the progression of the neuroinflammatory and neurodegenerative response to intracortical microelectrodes is effected by the removal of either TLR2 or TLR4 from the innate immunity process. Therefore, expression of immunohistochemical markers for neuronal survival, microglia and macrophage activation, astrocytic encapsulation, and blood brain barrier permeability were compared between the 2 week and 16 week time points for WT (Figure 4), *Tlr2*^−/−^ (Figure 5), and *Tlr4*^−/−^ mice (Figure 6), independent of each other.

#### The progression of neuroinflammation and neurodegeneration in WT mice

Changes in immunohistochemical markers between the acute 2-week and chronic 16-week time points in WT mice will indicate the standard progression of chronic neuroinflammatory mechanisms in response to implanted neural probes in mice. Examination of neuronal density via the NeuN stain in WT mice exhibited significantly higher neuronal density at 2 weeks after probe implantation in distance intervals between 150 and 300 μm from the probe hole, ^*^p < 0.05 (2 wk WT: *N* = 5; 16 wk WT: *N* = 7) (Figure 4A). Additionally, examination of the accumulation of inflammatory activated microglia and macrophages via CD68 expression indicated WT mice exhibit significantly higher CD68 expression at 2 weeks after probe implantation in distance intervals between 0 and 100 μm from the probe hole, ^*^p < 0.05 (2 wk WT: *N* = 6; 16 wk WT: *N* = 7) (Figure 4B). In contrast, examination of the chronic glial scar as a function of GFAP expression revealed WT mice exhibit significantly higher GFAP expression at 16 weeks after probe implantation in distance intervals between 0 and 200 μm from the probe hole, ^*^p < 0.05 (2 wk WT: *N* = 6; 16 wk WT: *N* = 7) (Figure 4C). Similar to microglia/macrophage activation, blood-brain barrier permeability as a function of IgG expression revealed WT mice exhibit significantly higher IgG expression at 2 weeks after probe implantation in distance intervals 0–50 and between 400 and 600 μm from the probe hole, ^*^p < 0.05 (2 wk WT: *N* = 6; 16 wk WT: *N* = 7) (Figure 4D).

#### The progression of neuroinflammation and neurodegeneration in *Tlr2^−/−^* mice

Examining the time course of immunohistochemical markers in *Tlr2*^−/−^ mice will identify potential effects of TLR2 removal on the standard progression of chronic neuroinflammatory mechanisms in response to implanted neural probes. Contrary to the trend in WT mice, examination of neuronal density in *Tlr2*^−/−^ mice exhibited significantly higher neuronal density at 16 weeks than 2 weeks after probe implantation in the distance interval 250–300 μm from the probe hole, ^*^p < 0.05 (2 wk *Tlr2*^−/−^: *N* = 7; 16 wk *Tlr2*^−/−^: *N* = 5); (Figure 5A). While this is a significant finding, it is likely not appreciable for electrode performance. Similar to the trend observed in WT mice, the accumulation of inflammatory activated microglia and macrophages in *Tlr2*^−/−^ mice exhibited significantly higher CD68 expression at 2 weeks than 16 weeks after probe implantation. However, the distance intervals with higher CD68 expression were between 500 and 600 μm from the probe hole, ^*^p < 0.05 (2 wk *Tlr2*^−/−^: *N* = 8; 16 wk *Tlr2*^−/−^: *N* = 5) (Figure 5B), which likely does not impact device performance. Similar to the trend observed in WT mice, examination of the chronic glial scar as a function of GFAP expression exhibited significantly higher GFAP expression at 16 weeks after probe implantation at distance intervals between 0 and 200 μm from the probe hole, ^*^p < 0.05 (2 wk *Tlr2*^−/−^: *N* = 8; 16 wk *Tlr2*^−/−^: *N* = 5) (Figure 5C). Astrocytic encapsulation increases over time in *Tlr2*^−/−^ mice in similar distance ranges as WT mice. As seen in WT mice, blood-brain barrier permeability as a function of IgG in *Tlr2*^−/−^ mice revealed significantly higher IgG expression at 2 weeks after probe implantation compared to 16 weeks after probe implantation at the distance intervals 0–50 μm from the probe hole, ^*^p < 0.05 (Figure 5D) (2 wk *Tlr2*^−/−^: *N* = 5; 16 wk *Tlr2*^−/−^: *N* = 5).

#### The progression of neuroinflammation and neurodegeneration in *Tlr4^−/−^* mice

Examining the time course of immunohistochemical markers in *Tlr4*^−/−^ mice will identify potential effects of TLR4 removal on the standard progression of chronic neuroinflammatory mechanisms in response to implanted neural probes. Unlike WT mice and *Tlr2*^−/−^ mice, examination of neuronal density and accumulation of inflammatory activated microglia and macrophages in *Tlr4*^−/−^ mice exhibited no significant differences between time points (2 wk *Tlr4*^−/−^: *N* = 5; 16 wk *Tlr4*^−/−^: *N* = 5) (Figures 6A,B). Similar to trends observed in WT and *Tlr2*^−/−^ mice, examination of the chronic glial scar in *Tlr4*^−/−^ mice exhibited significantly higher GFAP expression at 16 weeks than 2 weeks after probe implantation. Unlike WT and *Tlr2*^−/−^ mice, significantly higher GFAP expression only occurred in the distance interval 0-50 μm from the probe hole, ^*^p < 0.05 (2 wk *Tlr4*^−/−^: *N* = 4; 16 wk *Tlr4*^−/−^: *N* = 5) (Figure 6C). Similar to WT and *Tlr2*^−/−^ mice, blood-brain barrier permeability as a function of IgG expression in *Tlr4*^−/−^ mice exhibited significantly higher IgG expression at 2 weeks than at 16 weeks after probe implantation. Unlike WT and *Tlr2*^−/−^ mice, significant differences occurred over the distance intervals between 0 and 150 μm from the probe hole, ^*^p < 0.05 (2 wk *Tlr4*^−/−^: *N* = 4; 16 wk *Tlr4*^−/−^: *N* = 5) (Figure 6D).

## Discussion

This study sought to interpret the roles of TLR2 and TLR4 in the neuroinflammatory response to implanted intracortical microelectrodes. Overall, this study reveals that full removal of TLR4 results in reduced blood-brain barrier permeability at an acute (2 weeks) and chronic time points (16 weeks), and reduced neuronal survival at chronic time points, compared to WT animals. Further, microglia/macrophage activation and blood-brain barrier permeability significantly decreased from acute to chronic time points, whereas astrocytic encapsulation significantly increased from acute to chronic time points in WT mice. Mice lacking TLR2 or TLR4 exhibited similar trends in decreasing blood-brain barrier permeability and increasing astrocytic encapsulation, but significant changes in microglia/macrophage activation close to the electrode-tissue interface from 2 to 16 weeks post-implantation. The findings presented here introduce more questions regarding the role of innate immunity receptors in the neuroinflammatory response to intracortical microelectrodes.

The first major finding of this study indicated that knocking out TLR4 resulted in decreased blood-brain barrier permeability around implanted intracortical microelectrode at both acute and chronic time points. The blood-brain barrier is a network of endothelial cells with tight junctions that protects parenchymal brain tissue from neurotoxic molecules and infiltrating inflammatory cells. Damage to the blood-brain barrier following intracortical microelectrode implantation has been linked to poor recording performance (Saxena et al., 2013), potentially through neuronal damage, altered extracellular ionic concentrations, or propagation of inflammatory mechanisms (Jorfi et al., 2015). It is important to note that improved blood-brain barrier permeability alone may not lead to improved intracortical microelectrode performance. Blood-brain permeability in response to implanted neural probes may be related to TLR4 signaling on several levels: TLR4 signaling in endothelial cells, release of cytokines in response to TLR4 activation, and oxidative damage caused by factors released in response to TLR4 activation. An upregulation of TLR4 has been observed in vascular endothelial cells in response to renal ischemia reperfusion injuries, and an increase in co-localization of TLR4 and vascular endothelial cells was observed in response to subarachnoid hemorrhages (Zhao et al., 2014; Zhang et al., 2015). Ischemic injury has been tied to neurodegeneration around implanted intracortical microelectrodes (Kozai et al., 2014). It is possible that signaling of TLR4 on vascular endothelial cells in response to the implanted intracortical microelectrodes contributes to permeability of the blood-brain barrier.

Activation of TLR4 on secondary cells may also be responsible for increased permeability of the blood-brain barrier. In addition to vascular endothelial cells, neurons and microglia also exhibited elevated co-localization with TLR4 in response to subarachnoid hemorrhages (Zhang et al., 2015). Leow-Dyke et al. demonstrated that factors released by neurons conditioned with the TLR4 ligand lipopolysaccharide (LPS), including RANTES (CCL5), KC (CXCL1), tumor necrosis factor-α (TNFα), and IL-6, promoted the migration of neutrophils across an endothelial monolayer *in vitro*, and prior application of a TLR4 antagonist to the neurons significantly reduced this effect (Leow-Dyke et al., 2012). Additionally, activation of TLR4 on microglia may lead to activation of the inflammatory NFκB pathway, which can induce the release of pro-inflammatory cytokines, such as TNFα, IL-6, IL1-β (O'Neill and Kaltschmidt, 1997; Pineau and Lacroix, 2009). TNF-α and IL1-β have been shown to increase permeability of the blood-brain barrier (Ballabh et al., 2004). Bennett et al. recently detected upregulation of genes encoding pro-inflammatory cytokines paired with downregulation of genes encoding junction proteins of the blood-brain barrier at acute time points following intracortical microelectrode implantation (Bennett et al., 2018). Coincidentally, Bennett et al identified enhanced expression of TNFα, IL-6, and KC (CXCL1) following intracortical microelectrode implantation, potentially indicating a role of neuronal cytokine and chemokine release (Leow-Dyke et al., 2012; Bennett et al., 2018). Activation of TLR4 on microglia may also lead to the release of reactive oxygen species (Reed-Geaghan et al., 2009). Reactive oxygen species can promote leakiness of the blood-brain barrier (Merrill and Murphy, 1997; Lehner et al., 2011). In the absence of TLR4, less pro-inflammatory cytokines and reactive oxygen species are likely released, and thus less damage to the blood brain barrier occurs. Conversely, *Tlr4*^−/−^ mice did not exhibit any significant differences in microglia/macrophage activation. Differences in CD68 expression may not be sensitive enough to confer differences in cytokine and ROS release by activated microglia and macrophages, other infiltrating myeloid cells or neurons may be driving the release of factors damaging the blood-brain barrier, or TLR4 signaling on endothelial cells may facilitate blood-brain barrier permeability.

The next major finding of this study, indicating a reduction in neuronal survival around implanted intracortical microelectrodes in mice lacking TLR4, is more difficult to explain. Neurons are the source of signals recorded by intracortical microelectrodes and hypothesized to be needed within 50 μm of the microelectrode to record single units (Buzsáki, 2004). Although neuronal dieback has frequently been observed around implanted intracortical microelecrodes (Biran et al., 2005; McConnell et al., 2009; Potter et al., 2012a) and neuronal dieback has been hypothesized to cause intracortical microelectrode failure (Biran et al., 2005), the relationship between neuronal dieback and recording performance has not been fully elucidated (Jorfi et al., 2015). Typically, knocking out TLR4 results in neuroprotective effects (Tang et al., 2007; Hyakkoku et al., 2010). Here the opposite trend is observed. Perhaps the reduced capability to detect and fight pathogens makes mice more susceptible to localized infections; however, no indications of localized infection were seen in this study. Robust sterilization methods such as ethylene oxide sterilization do not always reduce endotoxin levels below the FDA requirement for devices implanted in the brain (Ravikumar et al., 2014a). Hermann et al. proposed that the reduced capability to detect and respond to tissue damage may hinder wound healing mechanisms beneficial to integrating devices into the brain (Hermann et al., 2018). Studies investigating the role of TLRs in neurodegenerative disorders such as Alzheimer's and synucleinopathies suggested that some amount of TLR signaling was necessary to clean up the accumulation of abnormal protein deposits (Tahara et al., 2006; Stefanova et al., 2011; Fellner et al., 2013) and neurons damaged by the proteins (Bate et al., 2004). Damaged matrix proteins and necrotic cells resulting from the implantation and chronic presence of an intracortical microelectrode that are normally recognized and disposed of by TLR4 mediated pathways may be detrimental to neurons directly or through the activation of redundant inflammatory mechanisms.

The third major finding of this study identified differences in the time course of the foreign body response to implanted intracortical microelectrodes in the absence of TLR2 and TLR4. *Tlr2*^−/−^ and *Tlr4*^−/−^ mice exhibited most of the same trends as WT mice between the 2 and 16 week time points, except for microglia/macrophage activation. WT mice exhibit a significant reduction in microglia and macrophage activation whereas *Tlr2*^−/−^ and Tlr4^−/−^ mice do not exhibit significant changes close to the electrode-tissue interface over time. The trend in WT mice indicate that TLR2 and/or TLR4 may play an important role in the activation of microglia and macrophages at the acute time point, despite the lack of significant differences between either *Tlr2*^−/−^ or *Tlr4*^−/−^ mice and WT mice. *Tlr2*^−/−^ or *Tlr4*^−/−^ mice both demonstrated lower peak CD68 intensities, but the intensities decayed to similar values over a short span of distance (~10 μm). Differences in CD68 expression may be limited to the first layer of cells around the neural probe hole. The decrease in CD68 expression in WT mice over time may indicate a diminishing importance of TLR2 and TLR4 in the chronic neuroinflammatory response to intracortical microelectrodes over time, or that lacking either TLR2 or TLR4 retards the rate of wound healing / scar progression. For example, TLR4 deficient mice exhibited delayed skin wound closure paired with decreased Il-1β and IL-6 production (Chen et al., 2013), indicating the importance of TLR4 activation and subsequent cytokine release in wound healing. Similarly, *Tlr2*^−/−^, *Tlr4*^−/−^, and *Tlr2/4*^−/−^ mice exhibited larger skin wound areas paired with a reduction in infiltrating macrophages and decreased expression of TGF-β and CCL5 (RANTES) (Suga et al., 2014). On the contrary, *Tlr2*^−/−^ and *Tlr4*^−/−^ mice exhibited improved wound healing in response to diabetic skin injuries (Dasu et al., 2010). Opposing outcomes of knocking out TLRs have been attributed to differences in acute and chronic injures, where chronic inflammation, as found in diabetic injuries, may hinder wound healing (Dasu and Jialal, 2013; Portou et al., 2015). The unresolved presence of an implanted intracortical microelectrode would likely behave like other chronic injuries. The role of TLR2 and TLR4 on wound healing in the brain would be difficult to predict, since activation of TLRs promotes injury or wound healing in a variety of injuries throughout the body (Kluwe et al., 2009), and the effects are hypothesized to be dose dependent (Strbo et al., 2014), timing dependent (Dasu and Rivkah Isseroff, 2012), and location dependent (Kluwe et al., 2009; Dasu and Rivkah Isseroff, 2012). In the context of spinal cord injury, *Tlr4*^−/−^ mice exhibited deficits in locomotor recovery and elevated demyelination, astrogliosis, and macrophage activation, and *Tlr2*^−/−^ mice exhibited deficits in locomotor recovery paired with abnormal myelin patterning (Kigerl et al., 2007). These findings suggest that TLR2 and TLR4 may play a beneficial role in the recovery of CNS injuries. However, the exact role of TLR2 and TLR4 in wound healing of the CNS remains to be elucidated.

Another interpretation of the time course of CD68 expression suggests that the enhanced CD68 expression in the WT group at 2 weeks post-implantation may be, in part, due to a higher infiltration of activated macrophages at the electrode tissue interface, considering the enhanced permeability of the blood-brain barrier at that time point. The contribution of infiltrating macrophages is important to consider since infiltrating macrophages have been shown to induce detrimental effects on neurons following central nervous system injuries (Horn et al., 2008; Busch et al., 2009). In contrast to the WT group, the *Tlr4*^−/−^ mice did not exhibit enhanced CD68 expression at 2 weeks post implantation and blood-brain barrier permeability was significantly lower than in WT mice at that time point. Lower BBB permeability at 2 weeks post-implantation may lead to less activated macrophage infiltration, resulting in CD68 expression comparable to 16 weeks post-implantation in *Tlr4*^−/−^ mice. On the other hand, *Tlr2*^−/−^ mice, which exhibited similar BBB permeability to WT mice did not exhibit elevated CD68 expression close to the electrode-tissue interface at 2 weeks after implantation. Regardless, assessing the contributions of microglia and macrophages require alternate methods, such as bone marrow chimeras with labeled blood-derived cells (Ravikumar et al., 2014b).

Comparing the findings of this study to previous studies in our lab, inhibiting the TLR co-receptor will provide a greater understanding of the role of innate immunity receptors in the chronic inflammatory response to intracortical microelectrodes. Hermann et al. previously observed that knockout mice lacking CD14 exhibited enhanced recording performance over the acute (0–2 weeks) but not chronic (2–16 weeks) time range, with no differences in neuronal survival, microglia/macrophage activation, astrocytic encapsulation, or blood-brain barrier permeability at 16 weeks after implantation (Hermann et al., 2018). Here we observe that knockout mice completely lacking TLR4 exhibit significantly reduced blood-brain barrier permeability at both acute and chronic time points. Although TLR4 and CD14 are closely associated in the recognition of ligands, knocking out TLR4 but not CD14 resulted in reduced blood-brain barrier permeability. Activation of TLR4 to promote blood-brain barrier permeability does not require CD14. TLR4 is able to bind and respond to its ligand LPS in the absence of CD14, although with drastically less sensitivity (Janova et al., 2015). There is evidence that TLR4 may bind and respond to DAMPs without CD14 (Allam et al., 2012), but the exact role of CD14 in the recognition of structurally diverse DAMPs by TLR4 remains to be elucidated. Further, knocking out TLR4 but not CD14 resulted in significantly lower neuronal survival (Hermann et al., 2018). It appears that fully removing TLR4 exhibits a beneficial effect at acute time points, as with CD14, but fully removing TLR4 at chronic time points is also detrimental. The presence of functioning TLR4 signaling may be more critical for long-term wound healing than CD14, since *Tlr4*^−/−^ mice exhibited significantly decreased neuronal survival and *Cd14*^−/−^ mice exhibited no differences from wildtype mice at the chronic time point. Alternatively, chronic decreases in neuronal survival in *Tlr4*^−/−^ mice may be a carry-over effect from improper wound healing at earlier time points. Experiments stopping and starting or delaying the administration of TLR4 antagonists to mice with implanted intracortical microelectrodes could potentially delineate the time-dependent role of TLR4 in the neuroinflammatory response. TLR4 and CD14 play related but independent roles that vary over time in the foreign body response to intracortical microelectrodes.

In addition to investigating the complete removal of CD14 via a knockout mouse, Hermann et al. observed that administering a small molecule inhibitor to the CD14-TLR4 complex improved recording performance at acute time points and out to chronic time points (16 weeks), without any differences in 16 week endpoint histology. Inhibition via a small molecule antagonist is less complete as full removal of a receptor via knockout. Residual TLR4 signaling from incomplete inhibition may protect neurons from the processes detrimental to neuronal survival in *Tlr4*^−/−^ mice at chronic time points after intracortical electrode implantation. The role of innate immunity signaling in the foreign body response to intracortical microelectrodes is dependent on the degree of receptor inhibition.

Further studies by Bedell et al. investigated the effects of knocking out CD14 in specific cell populations on intracortical microelectrode recording performance (Bedell et al., 2018). Mice with infiltrating blood-derived cells lacking CD14 and resident cells featuring intact CD14 exhibited significantly improved recording performance over wildtype mice over the 16-week study without any differences in 16-week endpoint histology. TLR4 signaling may have different roles on resident cells than on infiltrating myeloid cells. Since TLR4 is constitutively expressed in parenchymal microglia as opposed to CD14, knocking out TLR4 on resident or infiltrating cells only may produce vastly different results from CD14. The roles of innate immunity signaling are cell-specific.

Initially, we hypothesized that TLR2 and TLR4 play a role in the neuroinflammatory response to implanted intracortical microelectrodes. Although *Tlr2*^−/−^ mice did not exhibit any significant differences in endpoint histology, changes in blood-brain barrier permeability and neuronal survival in *Tlr4*^−/−^ mice would suggest a role of TLR4 in the neuroinflammatory response to intracortical microelectrodes. Differences in the time course of microglia/macrophage activation observed in *Tlr2*^−/−^ mice suggest a subtler role in the neuroinflammatory response to intracortical microelectrodes. Further, we proposed TLR2 and TLR4 signaling as a mechanism for the activation of microglia and macrophages and subsequent release of pro-inflammatory cytokines, ROS, and RNS in response to an implanted intracortical microelectrode. Here, we did not observe significant changes in microglia/macrophage activation via expression of CD68 in ether *Tlr2*^−/−^ or *Tlr4*^−/−^ mice at acute or chronic time points. However, the paradoxical decrease in blood-brain barrier permeability and decrease in neuronal survival observed in *Tlr4*^−/−^ mice may be affected by decreased or increased release of pro-inflammatory factors. As stated earlier, differences in expression of CD68 may not be sensitive enough to detect functional differences in cytokine, ROS, and NOS release. Alternatively, activation of TLR4 on other infiltrating myeloid cells not expressing CD68, endothelial cells, or neurons may be responsible for the observed histological changes (Ravikumar et al., 2014b). Future studies investigating the effects of innate immunity inhibition on gene and mRNA expression following the implantation of intracortical microelectrodes may elucidate changes in the production of cytokines, ROS, and NOS (He et al., 2006; Ereifej et al., 2018). Since this study employed intracortical microelectrode arrays without functional recording sites, conductive traces, and insulating layers, the effects of knocking out TLR2 and TLR4 on oxidative damage to these structures could not be determined via SEM. Much remains to be learned about the specific roles of TLR2 and TLR4 in the neuroinflammatory response to intracortical microelectrodes.

Building off of the previous studies of Hermann et al. and Bedell et al. innate immunity signaling pathways appear to play a role in the neuroinflammatory response against intracortical microelectrodes (Bedell et al., 2018; Hermann et al., 2018). The findings of this study suggest that fully removing TLR4 is beneficial at acute time points and fully removing TLR4 at chronic time points is detrimental. Based off the success of cell-specific CD14 inhibition exhibited by Bedell et al. (2018), incomplete inhibition of TLR4 and its co-receptors via small molecule antagonists or antibodies may be of further interest for improving the long-term performance of intracortical microelectrodes. Delaying or stopping and starting the administration of TLR4 inhibitors may be more appropriate to address the time variant role in the foreign body response to implanted intracortical microelectrodes. Targeting TLR4 on specific cell populations may be more beneficial than inhibiting TLR4 in the whole body. The Toll-like receptors and their adapter molecules affect the foreign body response in a time, method/extent of inhibition, and cellular subset –dependent manner. Future strategies to integrate intracortical microelectrodes using modulation of innate immunity signaling pathways should consider these parameters to optimize the preservation of electrode and tissue integrity.

## Conclusions

Complete removal of TLR2 via genetic knockout did not result in meaningful changes in the expression of the markers stained for in this study. Thus, TLR2 does not likely play a significant role in the foreign body response to intracortical microelectrodes. Complete removal of TLR4 via genetic knockout results in reduced blood brain barrier permeability in response to implanted neural probes at acute and chronic time points, as well as reduced neuronal survival around implanted neural probes. Benefits of fully removing TLR4 are overshadowed by detrimental effects on neuronal survival. Inhibition of TLR4 without complete removal of the pathway or for intermittent time courses may still be worth investigating as an intervention for improving intracortical microelectrode integration.

## Author contributions

JH, SMS, RM, and JRC contributed substantially to the conception or design of the work, analysis, and interpretation of data for the work, drafting, and revising the manuscript for important intellectual content, approved the final version to be published, and agreed to be accountable for all aspects of the work. SL, AS, CW, AA, JC, SmS, ShS, WT, and GP aided in the collection and analysis of histological data. JH, SL, AS, CW, AA, JC, SmS, ShS, WT, GP, SMS, RM, and JRC approved the final version to be published and agreed to be accountable for all aspects of the work.

### Conflict of interest statement

The authors declare that the research was conducted in the absence of any commercial or financial relationships that could be construed as a potential conflict of interest.
